# Strangulated Amyand’s hernia: management during the COVID-19 pandemic

**DOI:** 10.1093/jscr/rjab153

**Published:** 2021-04-30

**Authors:** Zeeshan Afzal, Robert O’Neill

**Affiliations:** Cambridge Oesophago-gastric Centre, Addenbrookes Hospital, Cambridge University Hospital NHS Foundation Trust, Cambridge CB2 2QQ, UK; Cambridge Oesophago-gastric Centre, Addenbrookes Hospital, Cambridge University Hospital NHS Foundation Trust, Cambridge CB2 2QQ, UK

## Abstract

Amyand’s hernia, presence of the appendix within an inguinal hernial sac, is a rare condition. We report a case of a 68-year-old woman who presented during the COVID-19 pandemic with an acute right groin pain due to a tender incarcerated inguinal hernia. Cross-sectional imaging confirmed an Amyand’s hernia. She proceeded to open appendectomy via the inguinal canal and primary suture repair of her inguinal hernia. Patient was discharged the following day. Surgical management of Amyand’s hernia varies depending on the resources, clinical findings and personal experience. In our opinion and experience, open hernia reduction, appendectomy and primary tissue repair repairs the most effective and appropriate approach especially during the COVID-19 pandemic.

## INTRODUCTION

Amyand’s hernia is defined as presence of the appendix within an inguinal hernia sac. This condition was first described in 1735 by Claudius Amyand, who operated on an 11-year-old boy with an incarcerated inguinal hernia containing a perforated appendix [1].

It is a rare condition and accounts for <1% of all inguinal hernias. Acute appendicitis within an Amyand’s hernia occurs in 10%. It predominantly occurs on the right side and is more common in children and men [2, 3].

To our knowledge, <200 cases have been described in the literature so far, and this is the first case reported in literature that was managed during the COVID-19 pandemic. Several surgical approaches have been described; however, for a strangulated appendix within right inguinal hernia sac, we propose an open inguinal approach, appendectomy and primary suture repair of the inguinal hernia as the most effective and appropriate approach especially during COVID-19 pandemic.

## CASE REPORT

A 68-year-old woman presented to the emergency department with 1-day history of an acute onset right groin pain and a tender swelling. She had a past medical history of osteoporosis and hypertension and was on alendronic acid and ramipril regularly. Otherwise, she was fit and well with no significant past surgical history. On clinical examination she had a tender incarcerated right inguinal hernia, but no signs of systemic illness or sepsis or COVID-19-related symptoms.

Her vital signs were normal, and she was afebrile. Her initial laboratory tests were normal including white cell count of 8.3 × 10^9^/l, C-reactive protein of 4 mg/l and lactate of 0.9 mmol/l.

**Figure 1:**
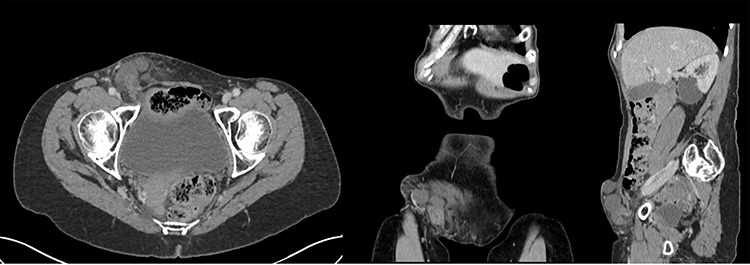
CT scan images of a 68-year-old woman who presented with acutely inflamed appendix within a right inguinal hernia sac (Amyand’s hernia) during COVID-10 pandemic.

She proceeded to computed tomography (CT) of her chest, abdomen and pelvis, which demonstrated acute appendicitis within a right, direct inguinal hernia (Amyand’s hernia) with no features of perforation, bowel obstruction or collections (Fig. 1).

There were no signs of COVID-19 on the chest CT. Her COVID viral swab was also negative.

**Figure 2:**
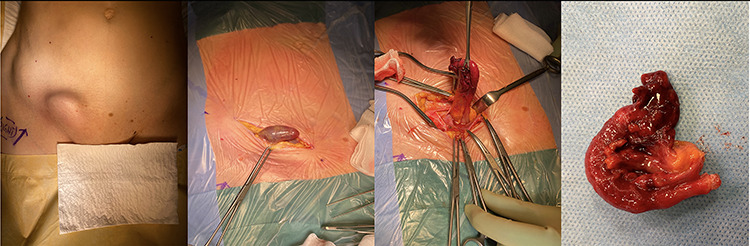
Intraoperative pictures of a 68-year-old woman who presented with acutely inflamed appendix within a right inguinal hernia sac (Amyand’s hernia) during COVID-10 pandemic: (**A**) Preoperative examination finding of right-sided incarcerated inguinal hernia. (**B**, **C**) Strangulated acute appendicitis. (**D**) Surgical specimen post-appendectomy.

She proceeded to surgery via an open inguinal approach. Intraoperative findings included a tight necked direct inguinal hernia containing strangulated distal appendix. The hernia sac contained haemoserous fluid only. An open appendicectomy was performed transfixing the appendiceal stump with 2/0 Ethicon Vicryl Suture. Although a mesh repair was considered, it was felt the risk of subsequent infection was too high, and a Bassini darn suture repair was performed with 1 Ethilon Nylon Suture; the external oblique aponeurosis was closed with 2/0 Ethicon prolene (Fig. 2).

The patient made an uneventful post-operative recovery and was discharged home the following day with 5 days of oral antibiotics. There were no signs of hernia recurrence after 3-month follow-up.

## DISCUSSION

To our knowledge, this is the first reported case of Amyand’s hernia managed during the COVID-19 pandemic. This rare hernia typically presents with groin pain and swelling and should be distinguished from a femoral or standard inguinal hernia. The appendix can be completed normal, inflamed, incarcerated or perforated [4]. Since all are indications for operative repair, the diagnosis is often made intraoperatively, although in many cases preoperative CT scanning can guide the operative approach or help determine operative risk in the case of concurrent COVID-19 infection [5–7].

Most surgeons would traditionally advocate appendectomy in the case of Amyand’s hernia, even if the appendix is normal, although this could be questioned in the light of recent data on the immunological function of the appendix. A further area of controversy is the use of mesh for hernia repair in a clean-contaminated procedure, and our preference in this case was to avoid mesh due to the risk of infection. Current guidelines advocate a laparoscopic approach for elective groin hernia repair in a woman, although in the acute setting with the likelihood of bowel resection open surgery is often still preferred. Laparoscopic repair has been reported for Amyand’s hernia [3, 8], although a further level of complexity was added due to the COVID-19 pandemic where national guidance suggested avoiding laparoscopic surgery. On this basis we proceeded with open surgery.

Losanoff and Basson described a systematic approach towards the management of Amyand’s hernia based on the appendix, which they classified from Type 1–4 (1 normal appendix, 2 uncomplicated appendicitis, 3 acute perforated appendicitis with peritonitis, 4 acute appendicitis with concomitant disease). For type 1, they recommended hernia reduction and mesh repair with or without appendectomy. For type 2, appendectomy, hernia repair without mesh. For type 3, appendectomy via midline laparotomy and hernia repair without mesh. Type 4 should be managed according to the underlying pathology [9]. In this case we followed these pragmatic recommendations.

## CONCLUSION

Amyand’s hernia is an uncommon condition. The majority of cases that presenting with acute appendicitis require appendectomy. The use of mesh in the repair remains controversial, but we advocate following the central principle that mesh should be avoided in a potentially contaminated field.

Several approaches towards surgical repair have been described in the literature; however, for a strangulated appendix within right inguinal hernia sac, we propose an open hernia reduction, appendectomy and primary tissue repair repairs as the most effective and appropriate approach especially during COVID-19 pandemic.

## CONFLICT OF INTEREST STATEMENT

None declared.
